# Composite Angioimmunoblastic T-Cell Lymphoma and Diffuse Large B-Cell Lymphoma Presenting with Distributive Shock

**DOI:** 10.3390/hematolrep15040064

**Published:** 2023-11-16

**Authors:** Nisha Hariharan, Alisha Kabadi, Michelle Don, Mazen Odish, Benjamin Heyman

**Affiliations:** 1 Department of Medicine, Division of Hematology/Oncology, University of California, La Jolla, CA 92093, USA; nishariharan@gmail.com; 2 Department of Medicine, Division of Pulmonary, Critical Care, Sleep Medicine, and Physiology, University of California, La Jolla, CA 92093, USA; 3 Department of Pathology, University of California, La Jolla, CA 92093, USA; 4 Department of Medicine, Division of Regenerative Medicine, University of California, La Jolla, CA 92093, USA

**Keywords:** angioimmunoblastic T-cell lymphoma, diffuse large B-cell lymphoma, composite lymphoma, brentuximab vedotin, shock

## Abstract

Diffuse large B-cell lymphoma (DLBCL) and angioimmunoblastic T-cell lymphoma (AITL) are two subtypes of non-Hodgkin lymphoma (NHL). The simultaneous occurrence of DLBCL and AITL in a composite lymphoma is very rare, and there are no established treatment regimens. We present the case of an 85-year-old male admitted to the intensive care unit with distributive shock, lymphocytosis, and lymphadenopathy, who was subsequently diagnosed with composite AITL and DLBCL, and treated with brentuximab vedotin (BV) and rituximab. To our knowledge, this is the first case of composite lymphoma presenting with distributive shock and treated with BV and rituximab, with successful resolution of shock.

## 1. Introduction

Non-Hodgkin lymphomas (NHLs) are classified by their cell of origin: B-, T-, or natural killer (NK)-cell. B-cell lymphomas comprise 85% of NHLs, with diffuse large B-cell lymphoma (DLBCL) being the most common [1,2]. NK/T-cell lymphomas comprise approximately 15% of all NHLs [1,2]. Angioimmunoblastic T-cell lymphoma (AITL) is a rare, aggressive peripheral T-cell lymphoma (PTCL) with a median overall survival of less than three years. It is characterized by intense inflammation and immune dysregulation [3]. Composite AITL and DLBCL is exceedingly rare, and its description has been limited to case series. It is still unclear how this composite lymphoma develops, but the presence of Epstein–Barr virus (EBV) in the setting of immune dysregulation from pre-existing AITL suggests that EBV-mediated B-cell lymphomagenesis may be the causative agent [3]. The optimal treatment remains uncertain. Here, we present a patient who was found to have a new diagnosis of composite AITL and DLBCL in the setting of distributive shock.

## 2. Case Presentation

An 85-year-old male with a history of coronary artery disease and heart failure with preserved ejection fraction was hospitalized for suspected heart failure exacerbation. He had a nine-year history of leukocytosis, and in the year prior to admission, developed peripheral eosinophilia and lymphadenopathy, with a previously non-diagnosed fine needle aspiration of a hilar lymph node.

On admission, initial labs were notable for worsened leukocytosis to 34,400/mm^3^ (absolute lymphocytes 6900/mm^3^, absolute eosinophils 1400/mm^3^), and Epstein–Barr virus (EBV) viral load of 1300 IU/mL (Table 1). Due to worsening leukocytosis and peripheral eosinophilia, Hematology was consulted. CT imaging demonstrated diffuse lymphadenopathy throughout the thorax, abdomen, and pelvis, the largest of which was a 2.4 cm right paratracheal lymph node. Bone marrow biopsy and excisional biopsy of two left iliac lymph nodes were obtained on hospital day (HD) 7. Eosinophilia work-up showed PDGFR-β rearrangement in 6–7% of peripheral blood cells. The work-up was negative for peripheral blood mutations in KIT, JAK2 (Janus Kinase 2), MPL (myeloproliferative leukemia), and CALR (calreticulin), and for Strongyloides immunoglobulin G (IgG). 

On HD8, the patient developed hypotension requiring two vasopressors and transfer to the intensive care unit. An extensive work-up for undifferentiated shock did not reveal an etiology. The infectious work-up showed only a right lower lobe lung opacity. Thoracentesis revealed transudative pleural fluid. The transthoracic echocardiogram was not consistent with cardiac eosinophilic infiltration. Despite empiric treatment with broad-spectrum antibiotics, there was no improvement in pressor requirements. 

**Figure 1:**
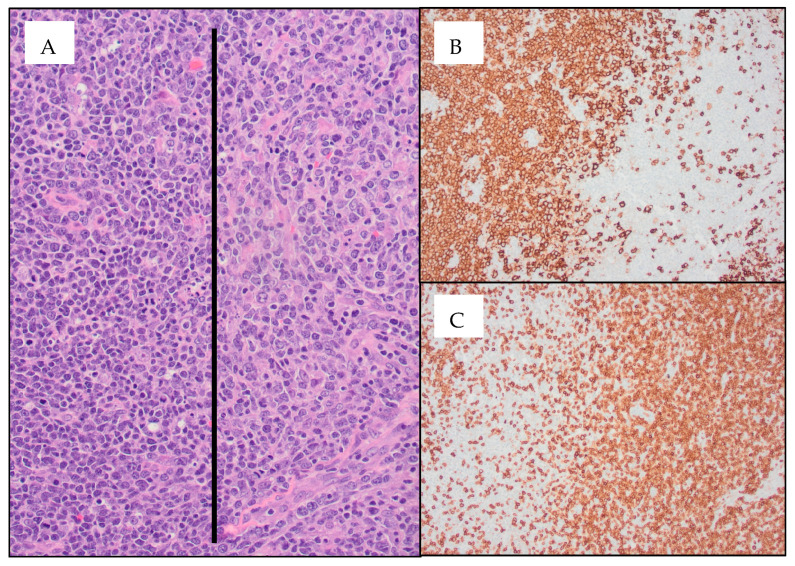
Diffuse large B-cell lymphoma (DLBCL) arising in the setting of angioimmunoblastic T-cell lymphoma (AITL) ((**A**) 20× image; AITL cells are seen to the right of the black line and DLBCL cells are seen to the left of the black line) within the same inguinal lymph node specimen. CD20 immunohistochemistry (IHC) stains highlight the DLBCL cells ((**B**) 10× image), which are seen next to the AITL cells highlighted using CD3 IHC stains ((**C**) 10× image). IHC was positive in AITL cells for CD2, CD3, CD4, CD5, CD10, CD45, BCL6, and PD1, with patchy CD30 staining of 15–20% of atypical cells. Focal involvement by large B-cells (germinal center phenotype) was positive for CD10, CD20, CD30, PAX5, BCL6, BCL2, MUM1, and EBER.

On HD14, the lymph node biopsy results returned, demonstrating composite lymphoma: AITL and DLBCL (Figure 1). T-cell receptor gamma gene rearrangement and gain of chromosome 3/3q were noted in both the bone marrow and the lymph node. IgH and IgK clonal rearrangements were noted in the lymph node alone. Bone marrow was involved by AITL but not DLBCL. 

Given persistent distributive shock with inability to wean vasopressors for seven days, suspicions increased that the cause of distributive shock was secondary to lymphoma. Methylprednisolone 1 mg/kg daily was initiated on HD14, and vasopressor requirements began to decrease. Given the patient’s age, shock status, and co-morbidities, he was deemed not to be a candidate for systemic chemotherapy, and thus brentuximab vedotin (BV) and rituximab were administered on HD20 and HD21, respectively. By HD22, all vasopressors were weaned off. He was discharged on HD31. The patient was recovering well at a one-week follow-up appointment, with plans for a second cycle of BV and rituximab. Unfortunately, the patient unexpectedly expired at home nineteen days after hospital discharge. 

## 3. Discussion

This is an atypical case of a new diagnosis of composite AITL/DLBCL presenting with distributive shock and responding to initial treatment with rituximab and BV. 

While multiple cases of composite AITL and DLBCL have been described, the pathophysiology of its development is not well-understood, though various hypotheses have been proposed. AITL is characterized by immune dysregulation which may lead to EBV reactivation and subsequent B-cell lymphomagenesis. In one study, 72% of cases with composite AITL and DLBCL were EBV-positive by in situ hybridization for EBER, suggesting that EBV plays a role in pathogenesis [4]. One suggested theory is that the development of AITL leads to EBV reactivation from dysfunctional, decreased T-cell immune surveillance. Because EBV preferentially infects B-cells [5], EBV-infected B-cells then proliferate in the absence of adequate immune surveillance [4,6,7]. This proposed mechanism is analogous to the pathogenesis of post-transplant lymphoproliferative disorders (PTLD), in which the iatrogenic immunosuppression of T-cells leads to the development of EBV-associated B-cell lymphomas [8,9].

It has also been proposed that AITL may develop by EBV-infected B-cells causing malignant transformation of T-cells. EBV-infected B-cells can activate T-cells via upregulation of the CD28 co-stimulatory ligand B7 found on B-cells. This upregulates T-cell expression of CXCL13, a B-cell chemokine, recruiting more B-cells to the affected lymph node, creating a co-stimulatory loop driving the development of both AITL and DLBCL [10]. It is notable that 3% of reported composite AITL and DLBCL cases are EBV-negative [6], suggesting that other pathogenic mechanisms may exist. For example, Fujisawa proposed that one of these mechanisms may include mutations in TET2, an enzyme involved in DNA methylation. TET2-mutated germinal B-cells may undergo clonal hematopoiesis and create a tumor microenvironment conducive to the clonal proliferation of T follicular helpers (TFH) cells, the cell of origin in AITL, leading to concurrent B- and T-cell lymphoma [11,12]. 

Our case is unique in its presentation of distributive shock. Shock as a presenting symptom of any NHL is uncommon. We identified 15 such cases in the literature (Table 2). It is notable that of these fifteen cases, six were PTCLs (three AITL, one PTCL NOS with AITL features, one PTCL NOS, and one anaplastic lymphoma). 

Proposed mechanisms for the development of shock include massive cytokine release from both malignant cells and activated macrophages, resembling cytokine storm [13,14,15]. Cytokine release may be further heightened in PTCL and AITL given the immune dysfunction prevalent in these lymphomas [15]. This is suggested by the increased proportion of PTCLs and AITL in cases of shock. Despite PTCL and AITL comprising 5–10% and 1–2% of all NHLs, respectively [2], they made up 40% and 20% of the fifteen NHL cases we identified presenting in shock. Systemic capillary leak syndrome (SCLS) should also be considered on the differential. While typically associated with paraproteinemia, there have been a small number of cases of PTCL in association with SCLS [16,17]. However, as these patients typically present with anasarca, this was considered less likely in our patient. 

Our patient’s clinical condition began to improve following the initiation of steroids, with the resolution of shock and weaning off all pressors after the administration of BV and rituximab, supporting that lymphoma was driving his shock state. 

There is little guidance on the optimal treatment of composite AITL and DLBCL given its rarity. Because most cases arise sequentially, patients are often first treated with an AITL-directed regimen, and following the development of DLBCL, a separate DLBCL-directed regimen. While rituximab, cyclophosphamide, doxorubicin, vincristine, and prednisone (R-CHOP) is the standard treatment for DLBCL, frontline treatment for AITL ranges from autologous stem cell transplant (SCT), to chemotherapy with CHOP, to immunosuppression. BV, cyclophosphamide, doxorubicin, and prednisone (BV-CHP) has also emerged as standard treatment for certain PTCLs such as systemic anaplastic large-cell lymphoma, though efficacy in other histologic subtypes such as AITL remains unclear [18]. Histone deacetylase (HDAC) inhibition may also have a role in AITL treatment, as HDAC inhibition has had efficacy in PTCL with the TFH phenotype, a disease entity similar to AITL with the same cell of origin [19]. 

In composite lymphoma, treatment is directed to the more aggressive lymphoma. For example, patients with concurrent AITL and indolent B-cell lymphomas such as SLL or FL were treated with CHOP-based regimens to target AITL [4,20]. Composite AITL and DLBCL poses a challenge since both lymphomas are considered aggressive, and treatment must take both into account. Of the 24 cases of composite AITL and DLBCL we identified in the literature, 14 received treatment, including rituximab, pirarubicin, cyclophosphamide, vincristine, and prednisone (R-THP-COP), R-CHOP, CHOP, R-fludarabine, auto-SCT, and thalidomide [4,7,21,22].

Patients presenting with shock pose a unique challenge given the concern that intensive chemotherapy may worsen their already critically ill state. In one case series, all eleven NHL patients in shock received chemotherapy, with or without rituximab, but only three patients survived the hospitalization [13]. In an effort to initiate conservative treatment given our patient’s multi-pressor requirement, we opted to treat initially with steroids, BV, and rituximab to target the respective T- and B- cell components of AITL and DLBCL. While this combination has been safely used in CD30+ B-cell lymphomas [23], to our knowledge, this is the only case of composite AITL and DLBCL treated with two immunotherapy agents targeting the respective T- and B-cells. While our patient had CD30 expression in over 10% of cells, studies suggest that BV efficacy may be independent of CD30 expression and could remain a treatment option even in CD30-negative PTCL [18,24]. Our patient was successfully weaned off vasopressors and discharged from the hospital soon after treatment. Our case suggests that rituximab and BV may represent a safe and well-tolerated alternative for patients, especially those in which critical illness is a concern, until further clinical improvement allows the addition of chemotherapy.

**Table 2:** Treatment and outcomes of Non-Hodgkin lymphoma patients presenting with shock.

Age/Sex	Diagnosis	Treatment	Outcome	Reference
35F	PTCL NOS	Antibiotics (lymphoma diagnosed post-mortem)	Died inpatient	[25]
70M	PTCL NOS (with features of AITL)	Stress-dose steroids	Off pressors within one day and discharged	[26]
59M	AITL	Stress-dose steroids	Died inpatient	[12]
73F	AITL	Steroids, alemtuzumab	Discharged	[13]
79F	CLL	CHOP	Died inpatient	[11]
53M	DLBCL	COP	Died inpatient	[11]
76M	DLBCL	R-CHOP	Died inpatient	[11]
79M	DLBCL	BR, followed by R-CHOP	Died inpatient	[11]
82F	DLBCL	CHOP	Discharged	[11]
68F	DLBCL with HLH	Etoposide	Discharged	[11]
58F	B-cell endovascular lymphoma	R-CHOP, etoposide	Died inpatient	[11]
56M	FL	COP	Discharged	[11]
78F	AITL	CHOP	Died inpatient	[11]
57F	T-cell lymphoma	Carmustine, cytarabine, etoposide, melphalan	Died inpatient	[11]
19F	T-cell anaplastic lymphoma	CHOP	Died inpatient	[11]

AITL, angioimmunoblastic T-cell lymphoma; BR, bendamustine, rituximab; CLL, chronic lymphocytic leukemia; DLBCL, diffuse large B-cell lymphoma; FL, follicular lymphoma; NOS, not otherwise specified; PTLC, peripheral T-cell lymphoma; R-CHOP, rituximab, cyclophosphamide, doxorubicin, vincristine, prednisone.

## 4. Conclusions

As more cases emerge describing composite AITL with B-cell lymphomas (most commonly DLBCL), it is important to quickly recognize and initiate appropriate treatment. In patients with hematologic abnormalities and persistent, undifferentiated shock, lymphoma should be considered on the differential. For composite lymphomas in critically ill patients, immunotherapy may represent a well-tolerated and effective initial treatment. 

## Figures and Tables

**Table 1:** Complete blood count at admission and discharge.

Lab Parameter	Day of Admission	Day of Discharge
WBC (1000/mm^3^)	34.4	15.0
RBC (million/mm^3^)	2.69	2.38
Hgb (g/dL)	9.7	8.3
Hct (%)	29.7	25.2
MCV (μm^3^)	110.4	105.9
RDW (%)	20.6	21.0
Plt (1000/mm^3^)	408	240
ANC (1000/mm^3^)	23.0	10.5
Lymphocytes (1000/mm^3^)	6.9	42
Monocytes (1000/mm^3^)	1.4	0.3
Eosinophils (1000/mm^3^)	1.4	0.0
Basophils (1000/mm^3^)	0.3	0.0

ANC, absolute neutrophil count; Hct, hematocrit; Hgb, hemoglobin; MCV, mean corpuscular volume; Plt, platelet; RBC, red blood cell; RDW, red cell distribution width; WBC, white blood cell.

## Data Availability

Additional information and data is available upon request.
